# Knowledge, attitudes, and willingness of bipolar disorder patients toward electroconvulsive therapy: a cross-sectional study

**DOI:** 10.3389/fpubh.2025.1572046

**Published:** 2025-06-25

**Authors:** Lin Zhou, Xinmeng Qi, Liuliu Xu, Xinrong Duanmu, Ke Wang, Kai Liu, Yue Zhang

**Affiliations:** ^1^Department of Psychiatry, The Affiliated Brain Hospital of Nanjing Medical University, Nanjing, China; ^2^Hospital Reform and Development Research Institute, Nanjing University, Nanjing, China

**Keywords:** knowledge, attitudes, and willingness, bipolar disorder, modified electroconvulsive therapy, health education, cross-sectional study

## Abstract

**Objective:**

This research aims to explore the levels of knowledge, attitudes, and willingness (KAW) of patients with bipolar disorder (BD) regarding electroconvulsive therapy (ECT).

**Methods:**

A cross-sectional survey was conducted in Nanjing from April 10 to November 3, 2024, using a validated questionnaire [Cronbach's α = 0.936, Kaiser–Meyer–Olkin (KMO) = 0.917]. Participants completed structured items assessing knowledge, attitudes, and willingness toward ECT. Data analysis involved descriptive statistics, non-parametric tests, Spearman correlation, multivariate logistic regression, and structural equation modeling (SEM).

**Results:**

The study successfully enrolled 479 participants. Of these, 282 participants (58.87%) were female. One hundred and sixty seven respondents (34.86%) had previously undergone ECT. The mean knowledge, attitude, and willingness scores were 5.57 ± 4.84 (possible range: 0–16), 29.08 ± 6.21 (possible range: 9–45), and 21.49 ± 5.14 (possible range: 6–30), respectively. SEM analysis showed that electroconvulsive therapy (β = −0.377, *P* = 0.014), years of BD (β = 0.196, *P* = 0.014) had direct effects on knowledge. Knowledge (β = 0.526, *P* = 0.023) directly affected attitude. Meanwhile, electroconvulsive therapy (β = −0.198, *P* = 0.013) and years of BD (β = 0.103, *P* = 0.016) indirectly affected attitude. Knowledge (β = 0.107, *P* = 0.018), attitude (β = 0.674, *P* = 0.009), and gender (β = 0.104, *P* = 0.020) directly affected willingness. Knowledge (β = 0.355, *P* = 0.011), electroconvulsive therapy (β = −0.174, *P* = 0.015), and years of BD (β = 0.090, *P* = 0.020) indirectly affected willingness.

**Conclusion:**

The study found that bipolar disorder patients generally lack knowledge and hold negative attitudes but demonstrate a relatively high willingness to accept ECT treatment. Targeted educational programs are recommended to improve understanding, shift attitudes, and enhance acceptance of this treatment in clinical willingness.

## Introduction

Bipolar disorder (BD) is a severe and chronic psychiatric condition characterized by recurrent mood episodes, including depressive episodes, manic or hypomanic episodes, mixed states, and inter-episodic periods of remission (1). Epidemiological studies estimate the lifetime prevalence of bipolar I disorder at ~1.06% and bipolar II disorder at 1.57% (2). Among these, depressive episodes are more commonly reported than manic episodes and are associated with a greater burden of disease (3, 4). Studies indicate that ~55.2% of patients experience a relapse within 2 years (5). Even during remission, patients often exhibit cognitive impairments, which significantly affect their daily functioning (6). While negative attitudes toward electroconvulsive therapy (ECT) are well-documented globally, regional variations in healthcare systems, cultural contexts, and treatment accessibility necessitate location-specific investigations. This study's focus on a Chinese metropolitan setting provides valuable insights into how local factors influence treatment perceptions and acceptance. Previous studies have predominantly been conducted in Western healthcare contexts, leaving a significant knowledge gap in understanding ECT attitudes within Asian healthcare systems, particularly in China's rapidly evolving mental health landscape. This regional perspective is crucial for developing culturally appropriate interventions to improve treatment acceptance and outcomes. Furthermore, BD is associated with increased suicide risk, higher rates of comorbidities, and accelerated physiological aging, all contributing to a markedly reduced life expectancy (7, 8).

Electroconvulsive therapy (ECT) which has been in use since the 1930s (9, 10). Advances in electrode placement have also been instrumental in optimizing outcomes, transitioning from bilateral to unilateral stimulation, typically on the right side (right unilateral, RUL). Additionally, the use of ultra-brief or brief square-wave pulse currents with individualized dosing strategies has been shown to minimize cognitive side effects (11–14).

While negative attitudes toward ECT are well-documented globally, regional variations in healthcare systems, cultural contexts, and treatment accessibility necessitate location-specific investigations (15). Previous studies have predominantly been conducted in Western healthcare contexts, leaving a significant knowledge gap in understanding ECT attitudes within Asian healthcare systems, particularly in China's rapidly evolving mental health landscape (16). The efficacy of ECT in treating bipolar disorder has been well-documented, particularly in cases of treatment-resistant depression, mania, and mixed states (17, 18). Clinical evidence suggests that significant symptom improvement, defined as a reduction of at least 50%, can be achieved within 2–3 weeks (approximately six sessions) of treatment (18). Moreover, studies have demonstrated that patients receiving ECT exhibit significantly lower all-cause mortality and suicide rates within 1 year post-discharge (19). Consequently, ECT is now recognized as a recommended therapeutic option in clinical guidelines for the management of bipolar disorder (20–23). It is important to note that ECT is not considered a first-line or second-line treatment for bipolar disorder. Rather, it is specifically reserved for severe cases such as treatment-resistant depression, severe manic episodes, and severe psychotic depression episodes (24, 25). The treatment can be administered either as acute intervention or maintenance therapy, with different protocols and considerations for each approach. In acute treatment, ECT is typically administered 2–3 times per week for 6–12 treatments, while maintenance ECT follows a more individualized schedule based on patient response and relapse prevention needs (26, 27).

The knowledge, attitudes, and willingness (KAW) model plays a pivotal role in understanding health-related behaviors and is frequently employed alongside KAW questionnaires to assess individuals' knowledge, attitudes, and willingness within healthcare contexts (28, 29). This theoretical framework posits a sequential relationship wherein knowledge positively influences attitudes, which subsequently shape willingness (30). As a cornerstone of health literacy, the KAW model also evaluates the acceptance and demand for specific healthcare interventions among target populations (28).

Despite its proven safety and efficacy, ECT remains underutilized in clinical willingness, largely due to persistent concerns among patients and the public. Common fears include apprehension about the procedure itself and potential memory impairments, both of which contribute to a reluctance to undergo this treatment (2, 31, 32). Such reservations are often rooted in a lack of understanding of the underlying mechanisms of ECT (32, 33).

Given these challenges, this study aims to investigate the KAW of patients with bipolar disorder to undergo ECT, thereby addressing gaps in understanding and potentially alleviating concerns surrounding its application.

## Materials and methods

### Study design and participants

This cross-sectional study was conducted in Nanjing from April 10, 2024, to November 3, 2024, involving patients diagnosed with bipolar disorder. Inclusion criteria: (1) had a diagnosis with bipolar disorder based on the International Classification of Diseases, 10th Revision, as confirmed by a physician qualified at the attending level or higher; (2) had sufficient cognitive ability and language skills to understand the questionnaire and communicate with the research team, as determined by a brief clinical interview; and (3) had provided informed consent voluntarily. Exclusion criteria: (1) the presence of comorbid psychiatric disorders; (2) severe visual or auditory impairments that would prevent understanding written or verbal instructions, even with corrective devices; and (3) inability to complete the questionnaire independently or with minimal assistance, based on a pre-survey evaluation by trained investigators. Ethical approval was obtained from the Clinical Research Management Committee of Nanjing Brain Hospital, and informed consent was secured from all participants prior to participation. Participants were recruited through convenience sampling from both outpatient clinics and inpatient wards at the Brain Hospital Affiliated to Nanjing Medical University. This sampling approach was chosen due to its feasibility and accessibility to the target population within our hospital setting. Participants were recruited through convenience sampling from both outpatient clinics and inpatient wards at the Brain Hospital Affiliated to Nanjing Medical University. This sampling approach was chosen due to its feasibility and accessibility to the target population within our hospital setting.

### Sample size calculation

We determined our sample size based on the well-established 5–10 events per variable (EPV) principle commonly used in survey research. Our questionnaire consisted of 24 structured questions (variables), which according to this principle would require a sample size of 120–240 participants (24 variables × 5 EPV = 120; 24 variables × 10 EPV = 240).

### Questionnaire introduction

The pilot study yielded 51 responses, of which 44 were deemed valid for analysis. The overall internal consistency of the questionnaire, as measured by Cronbach's α coefficient, was 0.889, with subscale coefficients of 0.930 for the knowledge dimension, 0.762 for the attitude dimension, and 0.881 for the willingness dimension. Additionally, the Kaiser–Meyer–Olkin (KMO) value for the pilot study was 0.955, reflecting excellent sampling adequacy. In the main study, a total of 479 valid questionnaires were collected. The overall Cronbach's α coefficient was 0.936, and the KMO value was 0.917, further demonstrating the questionnaire's strong reliability and construct validity. These metrics confirmed the robustness of the instrument for evaluating the targeted dimensions.

The knowledge, attitudes, and willingness (KAW) questionnaire was developed based on extensive literature review and expert consultation. The knowledge dimension assessed participants' understanding of ECT mechanisms, procedures, and effects through eight items covering two aspects: basic knowledge of ECT procedures and understanding of potential benefits and risks. The attitude dimension contained nine items evaluating emotional and cognitive responses toward ECT, including concerns about side effects, social stigma, and treatment efficacy. The willingness dimension comprised seven items measuring behavioral intentions and readiness to accept ECT treatment, considering both personal acceptance and compliance with medical recommendations. The finalized Chinese-language questionnaire consisted of four sections: (1) sociodemographic and clinical characteristics (including age, gender, residence, education level, employment status, monthly household income, marital status, duration of bipolar disorder, family history of mental health issues, history of electroconvulsive therapy, and type of medical insurance), (2) knowledge dimension, (3) attitude dimension, and (4) willingness dimension. The knowledge dimension comprised eight questions addressing two aspects of awareness. For the knowledge dimension, responses were scored as follows: “very familiar/correct” = 2 points, “heard of it/partially correct” = 1 point, and “unclear/incorrect” = 0 points. Higher scores indicated better understanding of ECT. For the attitude dimension, items were rated on a 5-point Likert scale from “very positive” (5 points) to “very negative” (1 point), with higher scores reflecting more positive attitudes. The willingness dimension used a similar 5-point scale from “strongly agree” (5 points) to “strongly disagree” (1 point), where higher scores indicated greater willingness to accept ECT. The attitude dimension included nine items rated on a five-point Likert scale, with options ranging from “very positive” (5 points) to “very negative” (1 point), yielding possible scores between 9 and 45. The willingness dimension consisted of seven items, six of which were scored on a similar five-point Likert scale from “strongly agree” (5 points) to “strongly disagree” (1 point), with total scores ranging from 6 to 30. Following established criteria from previous studies (15, 34, 35), scores exceeding 70% of the maximum possible score were categorized as indicative of adequate knowledge (>11.2 points), positive attitude (>31.5 points), or proactive willingness (>21 points). This categorization has been validated in similar healthcare assessment studies (36). In our sample, 272 participants (56.8%) scored above the willingness cutoff point of 21, while 207 participants (43.2%) scored at or below this threshold.

A combination of online and offline methods was employed for data collection. Paper-based questionnaires were distributed during outpatient clinic visits, while online surveys were administered through the Wenjuanxing platform. For participants who encountered difficulties in self-completion, trained members of the research team conducted face-to-face interviews to facilitate data collection. For the paper-based surveys, all investigators underwent standardized training to ensure adherence to the study protocol. This training included detailed guidelines on questionnaire distribution, as well as clarification of principles and precautions to ensure consistency in data collection. Two investigators were assigned to oversee data collection, ensuring its accuracy.

For the online component, a QR code linked to the electronic questionnaire was distributed through the “Mind Home” QQ group and displayed on ward bulletin boards. Participants accessed the survey using WeChat by scanning the QR code. To maintain data integrity, the system restricted submissions to one per IP address, and prompts alerted participants to address any unanswered items before submission. Entries with inconsistent or unreasonable responses were excluded. Nurses provided assistance to participants with online surveys as needed.

Data quality control was a key focus throughout the study. Investigators monitored the backend data in real time, and a double-entry method was utilized to ensure accuracy during data transcription. This multi-faceted approach addressed the diverse needs and capabilities of the participants.

### Statistical methods

Data analysis was performed using SPSS 27.0 (IBM Corp., Armonk, NY, USA) for statistical tests and AMOS 26.0 for structural equation modeling (SEM). Continuous variables were presented as means and standard deviations (SD), while categorical variables, including responses to specific questionnaire items, were expressed as frequencies and percentages. The statistical significance threshold was set at a two-sided *P*-value of <0.05. Group comparisons of knowledge, attitude, and willingness scores across demographic characteristics were conducted using non-parametric tests, as the KAW scores did not follow a normal distribution. For comparisons between two independent groups, the Mann–Whitney *U* test was applied, while the Kruskal–Wallis *H* test was used for comparisons among three or more groups. Spearman's rank correlation coefficient was employed to assess the relationships among the three dimensions of KAW, given the ordinal nature of the data. Independent risk factors associated with the willingness dimension were identified through multivariate logistic regression analysis. This method enabled the evaluation of the influence of knowledge and attitudes, alongside key demographic and clinical variables, on the likelihood of engaging in proactive health willingness. The model results were presented as odds ratios (ORs) with corresponding 95% confidence intervals (CIs). To explore the interrelationships among knowledge, attitudes, and willingness, structural equation modeling (SEM) was conducted. SEM allowed for the simultaneous examination of direct and indirect effects within the hypothesized KAW framework Model fit was evaluated using established indices, including the root mean square error of approximation (RMSEA), comparative fit index (CFI), Tucker–Lewis index (TLI), and incremental fit index (IFI). Models with RMSEA ≤ 0.08 and CFI, TLI, and IFI values ≥0.90 were considered acceptable.

## Results

### Demographic characteristics

Initially, a total of 589 samples were collected for this study. Samples with the following conditions were excluded, specifically: (1) 24 cases with a missing baseline information (one case who did not fill in the gender; six cases who did not fill in the age; one case who did not fill in the current work status; one case who did not fill in the marital status; two cases who did not fill in the diagnosed years; 10 cases who did not fill in the family history; two cases who did not fill in the electroconvulsive treatment status; and one case who did not fill in the type of medical insurance); (2) 56 cases whose age was <18 years; and (3) 27 cases with missing responses and three cases with abnormal responses to the KAW dimension; the final valid questionnaire was 479 cases.

Of the 479 participants, 282 (58.87%) were female, 247 (51.57%) were no more than 34 years old, 257 (53.65%) lived in urban areas, 143 (29.85%) had a Bachelor Degree or above, 315 (65.76%) were unemployed, 259 (54.07%) were single, 171 (35.7%) had been diagnosed with bipolar disorder for more than 3 years, 167 (34.86%) had received ECT. Significant differences in knowledge scores were found among participants with different demographic and clinical characteristics. Specifically, younger participants ( ≤ 34 years) had higher knowledge scores than older participants (6.19 ± 5.03 vs. 4.92 ± 4.56, *P* = 0.005). Urban residents scored higher than those in suburban and rural areas (6.09 ± 4.78 vs. 4.61 ± 5.05 and 5.14 ± 4.78, respectively, *P* = 0.005). Educational attainment was positively associated with knowledge scores, with the highest observed among those holding an associate degree (6.56 ± 5.68, *P* = 0.021). Participants with a longer duration of bipolar disorder (>3 years) had significantly higher scores (7.29 ± 5.24) than those with 1–3 years (4.80 ± 4.67) or <1 year of illness (4.50 ± 4.10; *P* < 0.001). Prior experience with ECT was also linked to markedly higher knowledge scores (8.77 ± 5.44) compared to those without such experience (3.66 ± 3.35; *P* < 0.001). Additionally, knowledge scores varied by type of medical insurance (*P* = 0.048), with participants holding commercial health insurance scoring highest (7.20 ± 4.89). Differences in attitude scores were more likely to be found among those with different family history of emotional disorders or other mental health issues (*P* = 0.044) and electroconvulsive therapy status (*P* < 0.001). Differences in willingness scores were more likely to be found among those with different gender (*P* = 0.026), monthly household income per capita (*P* = 0.045), family history of emotional disorders or other mental health issues (*P* = 0.024), and electroconvulsive therapy status (*P* < 0.001; Table 1).

**Table 1:** Demographic characteristics.

**Variables**	***N* (%)**	**Knowledge, mean ±SD**	***P*-value**	**Attitude, mean ±SD**	***P*-value**	**Willingness, mean ±SD**	***P*-value**
*N* = 479		5.57 ± 4.84		29.08 ± 6.21		21.49 ± 5.14	
**Gender**			0.943		0.958		**0.026**
Male	197 (41.13)	5.85 ± 5.39		29.08 ± 7.02		20.89 ± 5.21	
Female	282 (58.87)	5.38 ± 4.42		29.07 ± 5.59		21.91 ± 5.05	
**Age**			**0.005**		0.539		0.277
≤ 34 years old	247 (51.57)	6.19 ± 5.03		29.23 ± 6.29		21.83 ± 5.05	
>34 years old	232 (48.43)	4.92 ± 4.56		28.91 ± 6.14		21.13 ± 5.22	
**Residence**			**0.005**		0.349		0.892
Rural	155 (32.36)	5.14 ± 4.78		29.64 ± 5.60		21.81 ± 4.25	
Urban	257 (53.65)	6.09 ± 4.78		28.75 ± 6.44		21.24 ± 5.73	
Suburban	67 (13.99)	4.61 ± 5.05		29.01 ± 6.69		21.72 ± 4.59	
**Education**			**0.021**		0.954		0.579
Middle school or below	118 (24.63)	4.57 ± 4.69		28.84 ± 6.39		20.97 ± 4.90	
High school/technical school	125 (26.1)	5.70 ± 4.52		29.16 ± 5.67		21.51 ± 4.71	
Associate degree	93 (19.42)	6.56 ± 5.68		29.54 ± 6.28		21.88 ± 5.41	
Bachelor's degree or above	143 (29.85)	5.66 ± 4.54		28.90 ± 6.52		21.65 ± 5.52	
**Employment status**			0.917		0.608		0.291
Employed	164 (34.24)	5.45 ± 4.66		28.86 ± 5.65		21.88 ± 4.80	
Unemployed	315 (65.76)	5.64 ± 4.95		29.19 ± 6.49		21.29 ± 5.30	
**Monthly household income per capita**			0.207		0.228		**0.045**
<5,000	172 (35.91)	5.15 ± 4.64		28.89 ± 5.80		21.50 ± 4.82	
5,000–10,000	186 (38.83)	6.03 ± 4.97		29.66 ± 6.06		22.10 ± 4.95	
>10,000	121 (25.26)	5.47 ± 4.91		28.45 ± 6.95		20.55 ± 5.74	
**Marital status**			0.055		0.480		0.245
Single	259 (54.07)	5.93 ± 4.88		29.24 ± 6.37		21.77 ± 5.17	
Married	220 (45.93)	5.15 ± 4.77		28.88 ± 6.03		21.16 ± 5.09	
**Duration of bipolar disorder**			**<0.001**		0.822		0.366
<1 year	184 (38.41)	4.50 ± 4.10		28.78 ± 5.79		21.95 ± 4.75	
1–3 years	124 (25.89)	4.80 ± 4.67		29.12 ± 4.69		21.62 ± 4.60	
>3 years	171 (35.7)	7.29 ± 5.24		29.36 ± 7.51		20.91 ± 5.84	
**Family history of emotional disorders or other**			0.221		**0.044**		**0.024**
**mental health issues**							
Yes	139 (29.02)	6.05 ± 4.99		29.77 ± 6.08		22.24 ± 5.04	
No	340 (70.98)	5.38 ± 4.78		28.79 ± 6.26		21.19 ± 5.15	
**Undergone electroconvulsive therapy**			**<0.001**		**<0.001**		**<0.001**
Traditional electroconvulsive therapy	16 (3.34)	6.63 ± 5.19		29.50 ± 2.99		22.44 ± 5.97	
ECT	167 (34.86)	8.77 ± 5.44		32.48 ± 6.22		24.14 ± 4.25	
No	256 (53.44)	3.66 ± 3.35		26.72 ± 5.53		19.57 ± 4.88	
Don't remember	40 (8.35)	4.08 ± 2.76		29.78 ± 4.36		22.40 ± 4.65	
Only social health insurance	384 (80.17)	5.58 ± 4.86		29.42 ± 6.37		21.54 ± 5.22	
Only commercial health insurance	15 (3.13)	7.20 ± 4.89		27.67 ± 3.50		22.27 ± 5.30	
Both social and commercial health insurance	40 (8.35)	6.18 ± 4.35		27.80 ± 3.98		21.98 ± 3.86	
No insurance	40 (8.35)	4.33 ± 5.01		27.60 ± 6.96		20.28 ± 5.38	

Bold values indicate *P* < 0.05.

### Knowledge, attitude, and willingness dimensions

The mean knowledge, attitude, and willingness scores were 5.57 ± 4.84 (possible range: 0–16), 29.08 ± 6.21 (possible range: 9–45), and 21.49 ± 5.14 (possible range: 6–30), respectively. Based on the established criteria where scores exceeding 70% of the maximum possible score indicate adequate knowledge (>11.2 points), positive attitude (>31.5 points), or proactive willingness (>21 points), our findings reveal that patients generally demonstrated insufficient knowledge (mean score represents only 34.8% of the maximum possible score) and predominantly negative attitudes (mean score represents 64.6% of the maximum possible score) toward ECT. However, the willingness score (mean score represents 71.6% of the maximum possible score) exceeded the threshold for proactive willingness, with 272 participants (56.8%) scoring above the cutoff point of 21. The distribution of knowledge dimensions showed that the three questions with the highest number of participants choosing the “Unclear” option were “ECT may cause transient side effects such as elevated blood pressure or arrhythmias.” (K6) with 57.83%, “ECT requires a sustained treatment cycle to significantly alleviate symptoms of bipolar disorder.” (K8) with 56.78%, and “ECT may have positive effects on the brain and activate neuroplasticity.” (K3) with 50.52%. Responses to the attitude dimension showed that 16.08% strongly concerned and 41.75% concerned about social prejudice and misunderstandings related to ECT (A3), 25.05% strongly agreed and 33.4% agreed that Negative opinions from family and friends would make them resist accepting ECT (A4), and 34.86% strongly worried and 36.74% worried about the potential risks and side effects of ECT (A6). Overall, the responses to the attitude dimension indicate predominantly negative or concerned perspectives toward ECT, with a majority of participants expressing concerns about social prejudice (57.83%), negative family opinions (58.45%), and potential risks and side effects (71.6%). Across all nine attitude items, the average proportion of participants expressing negative or concerned responses was 53.7%, compared to 24.8% expressing positive or unconcerned responses, further supporting the conclusion that patients generally hold negative attitudes toward ECT. Responses to the willingness dimension showed that 16.08% disagreed and 7.31% strongly disagreed that they would actively consult doctors for information and advice about ECT (P2), 15.24% disagreed and 7.31% strongly disagreed that they would willing to consider ECT under the recommendation of healthcare professionals (P6), and 13.78% disagreed and 5.85% strongly disagreed that they would willing to participate in more ECT-related awareness and educational activities (P3; Supplementary Table S1).

### Correlations between KAW

Spearman correlation analysis indicated significant positive correlations between knowledge and attitude (*r* = 0.470, *P* < 0.001), as well as willingness (*r* = 0.452, *P* < 0.001). Meanwhile, there was also correlation between attitude and willingness (*r* = 0.746, *P* < 0.001; Table 2).

**Table 2:** Correlation analysis.

**Dimensions**	**Knowledge**	**Attitude**	**Willingness**
Knowledge	1		
Attitude	0.470 (*P* < 0.001)	1	
Willingness	0.452 (*P* < 0.001)	0.746 (*P* < 0.001)	1

### Univariate and multivariate logistic regression analysis

Multivariate logistic regression showed that knowledge (OR = 1.087, 95% CI: 1.017–1.161, *P* = 0.014), attitude (OR = 1.333, 95% CI: 1.252–1.420, *P* < 0.001), being male (OR = 0.593, 95% CI: 0.355–0.990, *P* = 0.046), and undergone ECT (OR = 1.840, 95% CI: 1.013–3.344, *P* = 0.045) were independently associated with willingness (Table 3).

**Table 3:** Univariate and multivariate logistic regression analysis on willingness.

**Variables**	**Univariate logistic regression**		**Multivariate logistic regression**	
	**OR (95% CI)**	***P*-value**	**OR (95% CI)**	***P*-value**
Knowledge	1.225 (1.165–1.288)	**<0.001**	1.087 (1.017–1.161)	**0.014**
Attitude	1.361 (1.285–1.442)	**<0.001**	1.333 (1.252–1.420)	**<0.001**
**Gender**				
Male	0.683 (0.473–0.986)	**0.042**	0.593 (0.355–0.990)	**0.046**
Female	Ref		Ref	
**Age**				
≤ 34 years old	1.175 (0.818–1.688)	0.382		
>34 years old	Ref			
**Residence**				
Rural	Ref			
Urban	0.887 (0.592–1.328)	0.559		
Suburban	0.749 (0.421–1.333)	0.326		
**Education**				
Middle school or below	Ref			
High school/technical school	1.354 (0.813–2.254)	0.244		
Associate degree	1.264 (0.730–2.189)	0.404		
Bachelor's degree or above	1.048 (0.642–1.708)	0.852		
**Employment status**				
Employed	1.403 (0.954–2.063)	0.085		
Unemployed	Ref			
**Monthly household income per capita**				
<5,000	Ref			
5,000–10,000	1.407 (0.921–2.148)	0.114		
>10,000	0.771 (0.484–1.229)	0.275		
**Marital status**				
Single	Ref			
Married	0.874 (0.608–1.256)	0.467		
**Duration of bipolar disorder**				
<1 year	Ref			
1–3 years	0.986 (0.622–1.562)	0.951		
>3 years	0.920 (0.604–1.400)	0.697		
**Family history of emotional disorders or other mental health issues**				
Yes	1.463 (0.975–2.195)	0.066		
No	Ref			
**Undergone electroconvulsive therapy**				
Traditional electroconvulsive therapy	1.370 (0.499–3.766)	0.541	0.511 (0.166–1.576)	0.243
ECT	5.565 (3.532–8.767)	**<0.001**	1.840 (1.013–3.344)	**0.045**
No	Ref		Ref	
Don't remember	1.675 (0.857–3.275)	0.132	0.760 (0.341–1.692)	0.501
**Type of medical insurance**				
Only social health insurance	1.201 (0.625–2.306)	0.583		
Only commercial health insurance	1.034 (0.315–3.396)	0.956		
Both social and commercial health insurance	1.357 (0.559–3.292)	0.499		
No insurance	Ref			

Bold values indicate *P* < 0.05.

### Interactions between KAW

**Figure 1:**
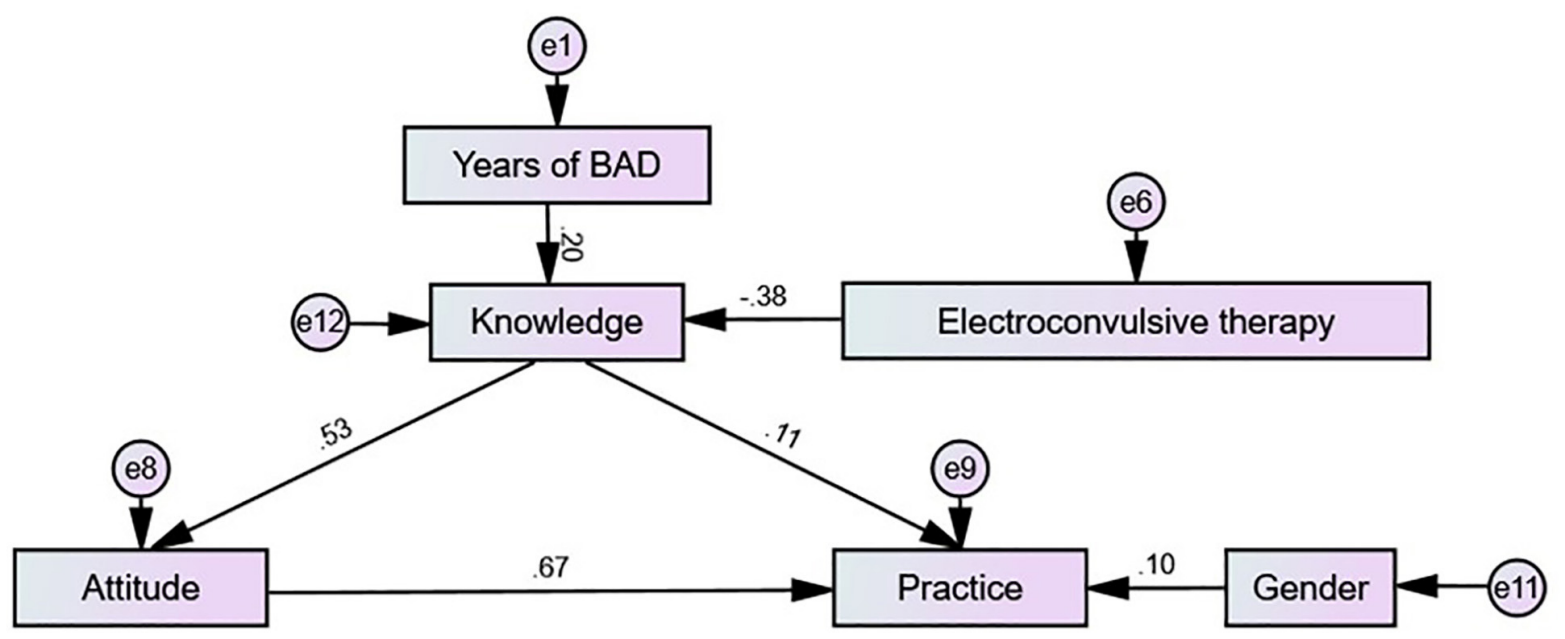
SEM analysis.

The SEM demonstrate a highly favorable model fit indices (GFI value: 0.960, RFI value: 0.843, IFI value: 0.917, and TLI value: 0.861), suggesting a well-fitting model (Supplementary Table S2). SEM analysis showed that electroconvulsive therapy (β = −0.377, *P* = 0.014), years of BD (β = 0.196, *P* = 0.014) had direct effects on knowledge. Knowledge (β = 0.526, *P* = 0.023) directly affected attitude. Meanwhile, electroconvulsive therapy (β = −0.198, *P* = 0.013) and years of BD (β = 0.103, *P* = 0.016) indirectly affected attitude. Knowledge (β = 0.107, *P* = 0.018), attitude (β = 0.674, *P* = 0.009), and gender (β = 0.104, *P* = 0.020) directly affected willingness. Knowledge (β = 0.355, *P* = 0.011), electroconvulsive therapy (β = −0.174, *P* = 0.015), and years of BD (β = 0.090, *P* = 0.020) indirectly affected willingness (Table 4 and Figure 1).

**Table 4:** SEM analysis.

**Model paths**	**Standardized total effects**		**Standardized direct effects**		**Standardized indirect effects**	
	**β (95% CI)**	***P*-value**	**β (95% CI)**	***P*-value**	**β (95% CI)**	***P*-value**
Electroconvulsive therapy → Knowledge	−0.377 (−0.447 to −0.311)	0.014	−0.377 (−0.447 to −0.311)	0.014		
Years of BD → Knowledge	0.196 (0.120–0.264)	0.014	0.196 (0.120–0.264)	0.014		
Electroconvulsive therapy → Attitude	−0.198 (−0.249 to −0.149)	0.013			−0.198 (−0.249 to −0.149)	0.013
Years of BD → Attitude	0.103 (0.058–0.148)	0.016			0.103 (0.058–0.148)	0.016
Knowledge → Attitude	0.526 (0.452–0.581)	0.023	0.526 (0.452–0.581)	0.023		
Electroconvulsive therapy → Willingness	−0.174 (−0.225 to −0.127)	0.015			−0.174 (−0.225 to −0.127)	0.015
Years of BD → Willingness	0.090 (0.050–0.125)	0.020			0.090 (0.050–0.125)	0.020
Knowledge → Willingness	0.462 (0.381–0.525)	0.023	0.107 (0.023–0.180)	0.018	0.355 (0.296–0.412)	0.011
Gender → Willingness	0.104 (0.030–0.159)	0.020	0.104 (0.030–0.159)	0.020		
Attitude → Willingness	0.674 (0.613–0.725)	0.009	0.674 (0.613–0.725)	0.009		

## Discussion

The study reveals that patients with bipolar disorder exhibit a significant gap in knowledge and generally hold negative attitudes toward ECT, although their willingness to undergo ECT is relatively high. Clinicians should focus on enhancing educational interventions that address misconceptions and provide comprehensive information about ECT, potentially increasing patient acceptance and adherence to recommended treatment protocols.

The results highlight critical challenges in patient understanding and perception of ECT, which are consistent with broader trends observed in mental health care. Previous research has indicated that misinformation and stigma surrounding electroconvulsive therapy often lead to apprehension and reluctance among patients, even when they recognize its potential effectiveness (37, 38). Similarly, our study found that knowledge deficits and misconceptions about the procedure are prevalent, reflecting a systemic issue in the dissemination of accurate and comprehensive information about ECT. This aligns with studies showing that inadequate patient education contributes to negative treatment attitudes, which can subsequently hinder adherence and therapeutic outcomes (39, 40).

The relationships among knowledge, attitudes, and willingness underscore the complexity of these challenges. Positive correlations between knowledge and attitude, as well as knowledge and willingness, emphasize the critical role of education in shaping patient perceptions. Path analysis further supports this relationship, demonstrating that knowledge directly influences attitudes and indirectly impacts willingness through improved attitudes. These findings are consistent with prior studies suggesting that patients with a better understanding of treatment options are more likely to perceive them positively and express willingness to engage in care (41, 42). However, the negative association between previous exposure to electroconvulsive therapy and attitudes highlights a paradox: while firsthand experience improves knowledge, it does not always translate to positive attitudes, potentially due to inadequate communication about the procedure's benefits and side effects during treatment (15, 36).

The influence of demographic and clinical factors further contextualizes these findings within broader health disparities. Younger patients, urban residents, and those with higher educational attainment were more likely to exhibit better knowledge and more positive attitudes. These patterns align with studies suggesting that access to educational resources and health literacy significantly impact patient understanding and perceptions of treatment options (43, 44). Moreover, patients who had previously undergone ECT showed higher levels of knowledge and willingness, indicating that direct exposure can demystify the procedure and reduce fear. However, concerns about social stigma, misinformation, and side effects remain barriers to acceptance, as reflected in both attitude dimensions and broader studies on mental health interventions (45, 46). The relatively limited impact of family history on attitudes in our study contrasts with findings from other research, where familial understanding of mental health treatments has been shown to positively influence perceptions (47, 48).

Knowledge dimension reveals a nuanced picture of patient awareness. Many participants demonstrated limited understanding of critical aspects of ECT, such as its role in neuroplasticity or the necessity of sustained treatment cycles. These findings mirror broader patterns in mental health care, where technical aspects of interventions are often poorly communicated to patients (49, 50). Socio-cultural factors and disparities in resource availability likely exacerbate these challenges. For instance, patients in rural or suburban areas exhibited lower levels of knowledge compared to their urban counterparts, reflecting systemic inequities in healthcare infrastructure and educational outreach. These patterns are consistent with studies highlighting the impact of geographic and socio-economic factors on access to mental health care and patient education (51, 52). Concerns about social stigma and potential side effects were particularly pronounced, underscoring the importance of addressing these misconceptions to improve acceptance of ECT.

These findings call for a multi-dimensional approach to improving patient understanding and acceptance of ECT. At a systemic level, healthcare organizations must prioritize equitable access to mental health education and resources, particularly in underprivileged and rural areas. Establishing integrated mental health education programs within community health centers could help bridge these gaps by providing targeted outreach and personalized education tailored to the needs of diverse populations. Specific efforts should include visual and interactive educational tools, such as videos and workshops, to enhance understanding of complex procedures like ECT (53, 54). These interventions should not only focus on the technical aspects of the treatment but also address common misconceptions and fears, using evidence-based communication strategies to improve patient confidence.

Healthcare professionals play a crucial role in this process and require adequate training to effectively communicate the benefits and risks of ECT. Incorporating ECT-specific modules into continuing medical education programs could enhance providers' ability to address patient concerns and build trust. Furthermore, leveraging patient testimonials and real-life case studies could serve as powerful tools for reducing stigma and fostering acceptance, as studies have shown that relatable narratives can significantly influence attitudes toward mental health treatments (35, 55). To support sustained improvements, it is essential to establish feedback mechanisms within healthcare systems to monitor the effectiveness of these initiatives and adapt them based on patient and provider input.

In addition to systemic and educational strategies, targeted policy changes are needed to address the structural factors contributing to disparities in ECT knowledge and attitudes. Policies that incentivize mental health education and awareness campaigns, particularly in underserved areas, could help reduce geographic and socio-economic inequities. Collaborative efforts between healthcare providers, policymakers, and community leaders are critical to ensuring that these initiatives are both culturally sensitive and contextually appropriate. For example, involving local mental health advocates in program design and implementation could enhance their relevance and effectiveness (56, 57).

This study has several limitations that should be considered when interpreting the findings. First, the cross-sectional design precludes any causal inferences regarding the relationships between knowledge, attitudes, and willingness related to ECT. Second, the use of self-reported questionnaires may introduce response biases, such as social desirability or recall bias, potentially affecting the accuracy of the data. Third, potential sampling bias may exist as participants were recruited through convenience sampling from a single hospital, which might not fully represent the entire bipolar disorder population. Fourth, although our questionnaire showed good reliability and validity in the pilot study, further validation with larger and more diverse populations would strengthen its psychometric properties. Fifth, as the study was conducted in a single city, the generalizability of the results to broader populations with bipolar disorder may be limited, necessitating further research in diverse geographic and cultural settings.

## Conclusion

In conclusion, patients with bipolar disorder demonstrated insufficient knowledge and predominantly negative attitudes toward ECT, despite showing a relatively high willingness to consider it as a treatment option. Tailored educational programs and counseling interventions that focus on improving patient understanding of ECT are essential to address misconceptions, enhance positive attitudes, and support informed decision-making, ultimately fostering greater acceptance and appropriate utilization of this therapeutic approach.

## Data Availability

The original contributions presented in the study are included in the article/Supplementary material, further inquiries can be directed to the corresponding author.
