# Supplementary material for: Knowledge, attitudes, and willingness of bipolar disorder patients toward electroconvulsive therapy: a cross-sectional study
Source: Front Public Health. 2025 Jun 25;13:1572046. doi: 10.3389/fpubh.2025.1572046 (PMC12237635; doi:10.3389/fpubh.2025.1572046)
Supplement: Supplementary file 1 [file Table_1.docx]

**Supplementary table 1. Knowledge, attitude and practice dimensions.**

| Knowledge | | **N (%)** | | | |
| --- | --- | --- | --- | --- | --- |
|  |  | **Very familiar** | | **Heard of it** | **Unclear** |
| 1. **Have you previously been aware that MECT can be used to treat bipolar disorder?** | | 89 (18.58) | | 219 (45.72) | 171 (35.7) |
| 1. **MECT is a physical therapy that involves anesthetizing the patient and relaxing muscles through injections, followed by stimulating specific cortical regions with external cranial electrical currents to induce "seizure-like discharges," achieving therapeutic effects.** | | 67 (13.99) | | 174 (36.33) | 238 (49.69) |
| 1. **MECT may have positive effects on the brain and activate neuroplasticity.** | | 76 (15.87) | | 161 (33.61) | 242 (50.52) |
| 1. **MECT can significantly improve patients' emotional states, reduce suicide risk, and lower hospital readmission rates.** | | 87 (18.16) | | 208 (43.42) | 184 (38.41) |
| 1. **Have you heard of successful cases of MECT helping patients with bipolar disorder?** | | 84 (17.54) | | 213 (44.47) | 182 (38) |
| 1. **MECT may cause transient side effects such as elevated blood pressure or arrhythmias.** | | 46 (9.6) | | 156 (32.57) | 277 (57.83) |
| 1. **MECT can improve the psychological health and cognitive function of patients with bipolar disorder.** | | 75 (15.66) | | 210 (43.84) | 194 (40.5) |
| 1. **MECT requires a sustained treatment cycle to significantly alleviate symptoms of bipolar disorder.** | | 74 (15.45) | | 133 (27.77) | 272 (56.78) |
| **Attitude** | **N (%)** | | | | |
|  | **Strongly agree** | **Agree** | **Neutral** | **Disagree** | **Strongly disagree** |
| 1. **I believe MECT is an effective treatment for bipolar disorder (P).** | 104 (21.71) | 181 (37.79) | 153 (31.94) | 31 (6.47) | 10 (2.09) |
| 1. **I believe MECT is a safe treatment for bipolar disorder (P).** | 91 (19) | 181 (37.79) | 102 (21.29) | 74 (15.45) | 31 (6.47) |
| 1. **I am concerned about social prejudice and misunderstandings related to MECT (N).** | 77 (16.08) | 200 (41.75) | 130 (27.14) | 45 (9.39) | 27 (5.64) |
| 1. **Negative opinions from family and friends would make me resist accepting MECT (N).** | 120 (25.05) | 160 (33.4) | 116 (24.22) | 57 (11.9) | 26 (5.43) |
| 1. **After learning more about MECT, I would consider undergoing this treatment (P).** | 92 (19.21) | 157 (32.78) | 133 (27.77) | 69 (14.41) | 28 (5.85) |
| 1. **I am worried about the potential risks and side effects of MECT (N).** | 167 (34.86) | 176 (36.74) | 106 (22.13) | 25 (5.22) | 5 (1.04) |
| 1. **I believe MECT can positively impact the quality of life of patients with bipolar disorder (P).** | 85 (17.75) | 192 (40.08) | 176 (36.74) | 23 (4.8) | 3 (0.63) |
| 1. **I think medical professionals should provide more education about MECT to increase patients' awareness (P).** | 128 (26.72) | 188 (39.25) | 132 (27.56) | 26 (5.43) | 5 (1.04) |
| 1. **I believe healthcare professionals performing MECT are professional and trustworthy (P).** | 159 (33.19) | 197 (41.13) | 105 (21.92) | 15 (3.13) | 3 (0.63) |
| **Practice** | **N (%)** | | | | |
|  | **Strongly agree** | **Agree** | **Neutral** | **Disagree** | **Strongly disagree** |
| 1. **I actively seek information about MECT through online media, such as articles or patient experiences (P).** | 77 (16.08) | 198 (41.34) | 120 (25.05) | 56 (11.69) | 28 (5.85) |
| 1. **I actively consult doctors for information and advice about MECT (P).** | 78 (16.28) | 191 (39.87) | 98 (20.46) | 77 (16.08) | 35 (7.31) |
| 1. **I am willing to participate in more MECT-related awareness and educational activities (P).** | 74 (15.45) | 162 (33.82) | 149 (31.11) | 66 (13.78) | 28 (5.85) |
| 1. **I discuss information about MECT with fellow patients (P).** | 89 (18.58) | 208 (43.42) | 121 (25.26) | 48 (10.02) | 13 (2.71) |
| 1. **(If applicable) I will promptly communicate with healthcare professionals and seek advice if discomfort or side effects occur during MECT (P).** | 158 (32.99) | 220 (45.93) | 80 (16.7) | 16 (3.34) | 5 (1.04) |
| 1. **I am willing to consider MECT under the recommendation of healthcare professionals (P).** | 102 (21.29) | 162 (33.82) | 107 (22.34) | 73 (15.24) | 35 (7.31) |
| 1. **Would you actively share your experience of undergoing MECT with others (only for patients who have received MECT N=167）** | 44 (26.35) | 79 (47.31) | 26 (15.57) | 18 (10.78) | / |

**Supplementary table 2. Model fit.**

| **Indicators** | **Ref** | **Results** |
| --- | --- | --- |
| GFI | >0.8 Good | 0.960 |
| RFI | >0.8 Good | 0.843 |
| IFI | >0.8 Good | 0.917 |
| TLI | >0.8 Good | 0.861 |
